# Studies on cellulase-ultrasonic assisted extraction technology for flavonoids from *Illicium verum* residues

**DOI:** 10.1186/s13065-016-0202-z

**Published:** 2016-09-14

**Authors:** Danna Huang, Xiaolei Zhou, Jianzhi Si, Xiaomei Gong, Shuo Wang

**Affiliations:** 1 Guangxi Botanical Garden of Medicinal Plants, Nanning, 530023 People’s Republic of China; 2 Guangxi Key Laboratory of Medicinal Resources Conservation and Genetic Improvement, Nanning, 530023 People’s Republic of China; 3 State Engineering Laboratory of Southwest Endangered Medicinal Resources Development, Nanning, 530023 People’s Republic of China

**Keywords:** Cellulase-ultrasonic, Extraction, Flavonoid, *Illicium verum*

## Abstract

**Background:**

*Illicium verum* is widely cultivated in southern China especially in Guangxi province. Its fruits has been traditionally used in Chinese medicine. In recent years, it has been the industrial source of shikimic acid. Usually the residues after extracting shikimic acid are treated as waste. Thus, the aim of this study was to optimize the extraction conditions of cellulase-ultrasonic assisted extraction technology for flavonoids from *I. verum* residues.

**Results:**

The optimum extraction conditions with a maximum flavonoids yield of 14.76 % are as follows: the concentration of ethanol is 51.14 %, the liquid–solid ratio is 20.52 mL/g, the enzymatic hydrolysis pH is 5.303, the sonication time is 60 min, the enzyme solution temperature is kept at 45 °C, the amount of added enzyme is 70 mg/g, the enzymatic hydrolysis time is 2 h and the crushed mesh size is 0.355–0.85 mm.

**Conclusions:**

The data indicate that the cellulase-ultrasonic assisted extraction technology has the potential be used for the industrial production of flavonoids from *I. verum*.

## Background

*Illicium verum* Hook. f., known as Chinese star anise, is a magnoliaceae evergreen arbor plant that grows mainly in Southwest China, especially in the provinces of Guangxi, Guangdong, Yunnan and Fujian. China is already the world’s largest producer of *I. verum*, with its cultivation as a medicinal plant in the Guangxi province accounting for approximately 90 % of the total output [1–3]. As a kind of popular cooking spice, the dried fruits of *I. verum* have also been used traditionally in Chinese medicines. In 2002, *I. verum* was categorized as both food and medicine by the Ministry of Health, People’s Republic of China and it is listed in the Chinese Pharmacopoeia with the actions of warming yang and dispelling cold, and regulating the flow of Qi to relieve pain [4–6]. The most valuable part of *I. verum* is the essential oil extracted from it which has a wide range of commercial applications including the production of perfumes, cosmetics, soaps, foods and beverage flavoring [7, 8]. Furthermore, *I. verum* is the industrial source of shikimic acid, a key intermediate used in the production of Tamiflu, which is a well-known antiviral drug and has recently been used to reduce the effects of bird flu [9]. *I. verum* has also been reported to possess antioxidant and antimicrobial activities due to its high concentrations of phenol compounds, and it is known that flavonoids also play an important role in this regard [10–12].

Medicinal plant material is used in a large number of phytopharmaceutical industries but the growing demand for these medicines means that the medicinal plant sources might no longer be capable of providing enough material in the future. However, the rich extracts from the *I. verum* biomass have traditionally been considered as waste because of inefficient extraction and separation processes [3], and usually the residues are treated as waste. A great number of innovative extraction methods such as ultrasound-assisted extraction, supercritical fluid extraction, extrusion and microwave extraction are now employed in the food industry [8]. Enzyme-assisted extraction is a mild, efficient and environmental friendly extraction method and it has been adopted for extracting various kinds of compounds recently [13]. The ultrasound-assisted extraction technique causes collapse of cavitation bubbles which generates sufficient energy to give rise to collisions between suspended plant particles for accelerating the release, diffusion and dissolution of active substances in the cell. On the other hand, enzyme-assisted extraction uses enzyme preparations either alone or in mixtures that catalyze hydrolysis of the cytoderm and glycoproteins, and enhance the release of bioactive substances by disrupting plant cells [14]. Enzymolysis-ultrasonic assisted extraction is a combined extraction method, which has advantages of the two extraction methods such as mild extraction conditions, lower investment costs and energy requirements, and simplified manipulation [15].

Recently, response surface methodology (RSM), which is a statistical technique to determine the influences of individual factors and their interactive influences, has been used increasingly to optimize processing parameters [8, 16–18]. In some previous reports, the optimization studies on enzymolysis-ultrasonic assisted extraction of *Cucurbita moschata*, *Lycium barbarum*, *Momordica charabtia*, wheat bran and corn silk have been performed using RSM [13–15, 19, 20]. Hence, the cellulase-ultrasonic assisted extraction technology for flavonoids from plants, combining the mild bio-enzymatic hydrolysis conditions and the rapid ultrasonic extraction technology, will protect the maximum bio-activity of the flavonoids. In this paper, we studied the optimization of cellulase-ultrasonic assisted extraction for flavonoids from *I. verum* residues using response surface methodology. The adsorption conditions were optimized from a single factor and orthogonal design experiments and desorption conditions were optimized from dynamic desorption experiments.

## Experimental

### Materials

The dried fruits of *I. verum* Hook. f. were collected from Baise County, Guangxi, China. A voucher specimen of this material was deposited in the herbarium of the Guangxi Botanical Garden of Medicinal Plants.

Cellulase was purchased from Sigma Company (USA, No. SC118401). Rutin used as the control was obtained from Sinopharm Chemical Reagent Co., Ltd (China, No. U1606503). Ethanol, methanol, petroleum ether were bought from Guangdong Xilong Chemical Factory (China). Hydrochloric acid was from Shanghai Ailian Chemical Reagent Company (China) and sulfuric acid was from Tianjing Qingfa Chemical Factory. Other reagents used in the experiments were purchased from Sinopharm Chemical Reagent Co., Ltd (China).

### Methodologies

#### Sample preparation

1 kg dried plant materials was powdered with a mill (Fz-02, Zhejiang Baile Mill Factory). After drying at 60 °C for 12 h, 0.9 kg of crushed material was used for extracting shikimic acid by a water extraction method [21], and then the residues were degreased and decolorized in petroleum ether with a ratio of 1:3 (m/v) at 60 °C, and carried on a backflow for 4 h. Subsequently, the residue product was dried at 60 °C for 48 h and weighed, then stored in a desiccator in order to maintain a constant weight for use in the subsequent experiments. The weight of the residue product was 0.8865 kg, which accounted for 98.5 % of the raw crushed materials.

#### Identification of flavonoids

An appropriate amount of the prepared samples was reflux with 80 % ethanol at a solid liquid ratio of 1:10 (m/v) at 80 °C for 2 h. The ethanol solution was concentrated by reducing the pressure and dried by vacuum. The total flavonoid extracts obtained were dissolved in methanol and then tested using the HCl–Mg reaction and the aluminum chloride colorimetric methods. The extracts responded positively to these characteristic color reactions for flavonoids.

#### Standard curve preparation

5.0 mg of rutin was dissolved in 60 % ethanol to a concentration of 0.2 mg/mL for use as the rutin standard solution. A set of standard solutions containing 0, 0.08, 0.16, 0.24, 0.32, 0.4 and 0.48 mg of rutin were made up in a total of 5 mL of 60 % ethanol. 0.4 mL of 5 % NaNO_2_ solution was added to each tube, which was incubated and shaken for 6 min, and then 0.4 mL of 5 % Al(NO_3_)_3_ solution was added and shaken for a further 6 min. 4 mL of 4 % NaOH solution was added and this was made up to 10 mL with 60 % ethanol. After incubation for 15 min, the rutin standard solutions with extracted flavonoids were developed by addition of a Na_2_NO_2_–Al(NO_3_)_3_–NaOH coloration system. This was the read at the wavelength range of 200–700 nm on an ultraviolet spectrophotometer. The absorbance was measured at 500 nm which is the selected maximum absorption wavelength and a standard curve was created.

#### Determination of optimum conditions for extraction of flavonoids

1.000 g of the prepared residue samples was soaked with 5 mL cellulose in a 50 mL centrifuge tube and citrate buffer was used to adjust pH. The enzymatic hydrolysis was conducted at a constant temperature and pH for several hours. After inactivating the cellulose at 100 °C for 5 min, 15 mL ethanol was added and the mixture was subjected to ultrasonic treatment. The extraction process was designed with these corresponding conditions. Using the following conditions of 10 mg/mL cellulase, 50 % ethanol, a mesh size of 0.355–0.85 mm, 2 h of enzymatic hydrolysis at pH 5, a liquid–solid ratio of 20:1, 60 min of sonication time and 40 °C, extraction temperature, the extraction yield of flavonoids from the *I. verum* residues was determined. Each of the parameters was kept as above while the others were varied as follows: cellulase concentrations (2, 6, 10, 14 and 18 mg/mL), ethanol concentrations (10, 15, 20, 25, 30 and 50 %), mesh sizes (0.85–2.0, 0.355–0.85, 0.25–0.355 and 0.18–0.25 mm), enzymatic hydrolysis times (0.5, 1, 1.5, 2, 2.5 and 3 h), pH (3, 4, 5, 6 and 7), different liquid–solid ratios (5:1, 10:1, 15:1, 20:1, 25:1 and 30:1), sonication times (15, 30, 45, 60, 75 and 90 min) and extraction temperatures (35, 40, 45, 50, 55, 60 and 65 °C). All tests were carried out in triplicate.

### Response surface optimization design

#### Determination of main experimental factors

On the basis of the single factor determinations of extraction experiments, we selected a set of experimental factors. The main experimental factors were further subjected to selection by the Plackett–Burman design in order to simplify the subsequent response surface experimental design.

#### Optimization by Box–Benhnken design

According to the principles of the Box–Benhnken design, the main experimental factors that affect the extraction process of flavonoids from residues of *I. verum* samples were optimized and the response surface analysis was carried out. The relationship between the extraction yield and each factor was established.

### Data analysis

Results were analyzed in triplicate and expressed as mean ± standard deviation. The data were analyzed by DPS statistical software and *p* < 0.05 was considered to be statistically significant.

## Results and discussion

### Standard curve and regression equation

**Fig. 1:**
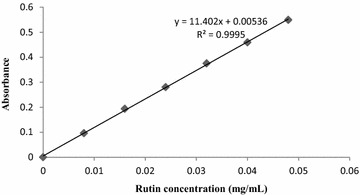
Standard curve

The absorbance was measured at the wavelength of 500 nm and the standard curve of rutin is shown in Fig. 1. Using the linear least square approach, the regression equation between the concentration and absorbance of rutin standard solutions was obtained as, A = 11.402C + 0.00536 (R^2^ = 0.9995). Its linear range is from 0.008 to 0.048 mg/mL. The linear calibration was performed to enable quantification of the flavonoids. The extraction yield of flavonoids from the samples was calculated as the percentage of the content according to the following equation:

$${\text{Extraction yield }}\left( \% \right) = \frac{C \times V}{m} \times 100\,\%$$

where *A* is absorbance, *C* is concentration of flavonoids (mg/mL), *V* is volume of solution (mL), and *m* is content of the test sample (mg).

### Single factor experiment

#### Effect of sonication time on the extraction yield

**Fig. 2:**
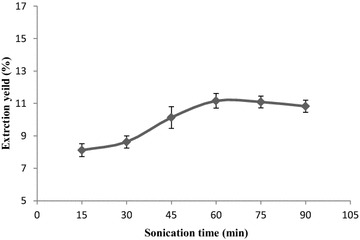
The effect of sonication times on the extraction yield of flavonoids

Starting from 15 min, the extraction yield increased with an increase of sonication time and reached the maximum value of 11.16 % at 60 min, and then it decreased gradually (Fig. 2). This may be due to low stability of some flavonoids which degraded due to ultrasonic heat effects, and may further result in destruction of their basic structures. The optimum ultrasonic processing time for extracting the flavonoids was determined to be 60 min.

#### Effect of liquid–solid ratio on the extraction yield

**Fig. 3:**
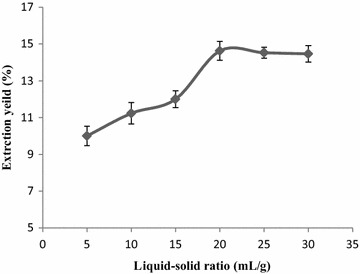
The effect of liquid–solid ratios on the extraction yield of flavonoids

Figure 3 reveals that the extraction yield of flavonoids was raised with an increased amount of the extracting agent initially from 5 mL and reached the maximum value of 14.63 % at the liquid–solid ratio of 20:1 after which it levelled off. When the liquid–solid ratio was too low, the flavonoids cannot be extracted adequately from the lysed cells. However, when ratio is too high, this will weaken the effect of ultrasonic waves on the fragmentation of the sample. Therefore, the liquid–solid ratio of 20:1 was selected.

#### Effect of ethanol concentration on the extraction yield

**Fig. 4:**
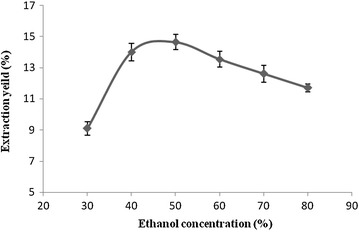
The effect of ethanol concentrations on the extraction yield of flavonoids

The effect of ethanol on flavonoids extraction was investigated from 30 to 80 %. It is seen in Fig. 4 that different concentrations of ethanol have an impact on the flavonoids extraction yield. Low or high concentrations of ethanol are not conducive to the optimum extraction and this is related to the type of flavonoids present in the sample. The flavonoid glycoside components with less polar portions are soluble in ethanol, however, those that are more polar are soluble in water. So when the ethanol concentration reaches the correct proportion, the total flavonoids extraction yield reached the highest value. The experimental results showed that 50 % ethanol was the optimal concentration to extract the flavonoids from the samples.

#### Effect of enzymatic hydrolysis pH on the extraction yield

**Fig. 5:**
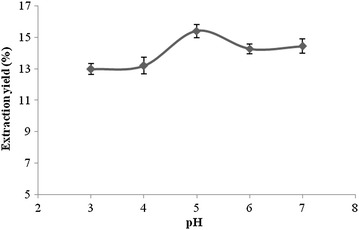
The effect of pH on the extraction yield of flavonoids

Figure 5 shows that in the range of pH from 3 to 7, the extraction yield was raised below pH 5 and reached the maximum extraction yield of 15.42 % at pH 5 and then it declined as it neared a neutral pH. This can be explained by the fact that the enzymatic hydrolysis pH had an effect on the cell wall with respect to the hydrolysis of cellulase. Reaction conditions with too much acid or alkali will lead to a loss of biological enzyme activity, and also increase the loss of other non-flavonoids ingredients, so those conditions are not conducive to the extraction of the target components.

#### Effect of extraction temperature on the extraction yield

**Fig. 6:**
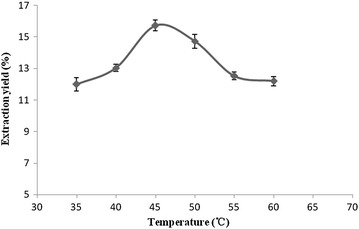
The effect of temperatures on the extraction yield of flavonoids

Figure 6 shows that from 35 to 45 °C, the flavonoids extraction yield increased with a rise of the enzymatic extraction temperature, and the peak yield (15.73 %) was reached at an extraction temperature of 45 °C. When the extraction temperature was higher than 45 °C, the extraction yield kept on decreasing due to the fact that the high extraction temperature always resulted in an increase of enzyme activity [19, 20]. Therefore, it can be considered that the optimum reaction temperature for enzymatic hydrolysis of the cell wall of *I. verum* is at 45 °C.

#### Effect of cellulase concentration on the extraction yield

**Fig. 7:**
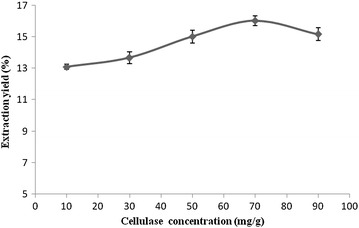
The effect of concentrations of cellulase on the extraction yield of flavonoids

It is seen in Fig. 7 that the flavonoids extraction yield increased with a rise in the concentration of cellulase. When the concentration was higher than 70 mg/g, the extraction yield dropped instead of increased. This means the cellulase concentration of 70 mg/g is high enough, and a higher concentration up to 70 mg/g did not further improve the extraction yield. It may be the viscous enzyme solution with high concentrations of cellulase is not conducive to the enzymatic reaction process.

#### Effect of enzymatic hydrolysis time on the extraction yield

**Fig. 8:**
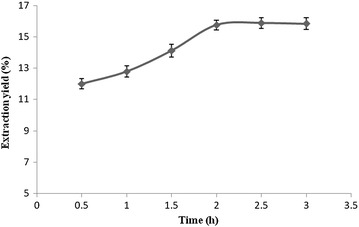
The effect of times of enzymatic hydrolysis on the extraction yield of flavonoids

It can be seen in Fig. 8 that the extraction yield is increased with a longer time of cellulase enzymatic hydrolysis, while after 2 h, the extraction yield levelled off. Therefore it seems that the enzymatic hydrolysis reaction completely destroyed the cell wall of the sample and released the maximum flavonoids composition within 2 h of incubation.

#### Effect of crushed mesh size on the extraction yield

**Fig. 9:**
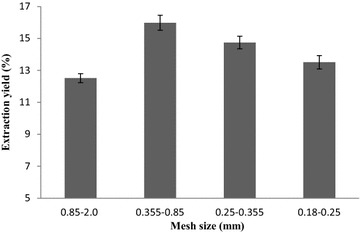
The effect of crushed mesh sizes on the extraction yield of flavonoids

As shown in Fig. 9, the crushed mesh size 0.355–0.85 mm is the optimum one to use under these conditions. It can be seen that improving the ability to crush raw materials will lead to an increased rate of flavonoids extraction, while high degrees of crushing will reduce the extraction yield. It might be that high degrees of grinding the raw materials will make the samples become more prone to stick into small groups and this may not be conducive to the enzymatic hydrolysis reaction and subsequent ultrasound extraction.

### Response surface methodology and results analysis

#### Plackett–Burman design and data analysis

Plackett–Burman design and data analysis can be seen in Tables 1 and 2. The individual factors of sonication time (A), liquid–solid ratio (B), ethanol concentration (C), pH (D), temperature (E), cellulase concentration (F), enzymatic hydrolysis time (G) and extraction yield (Y) were used. As shown in Table 2, the *p* value of the regression model is <0.05, which indicates that the results from the model are significant. For each of the experimental factors, the level of significance was C, B, D, F, A, E and G in that order. Since the ethanol concentration (C), liquid–solid ratio (B) and pH (D) have higher significant effects than the other factors, they were selected as the main optimization experimental factors for further response surface analysis.

**Table 1:** Scheme and experimental results of Plackett–Burman design

No.	A (min)	B (mL/g)	C (%)	D	E (°C)	F (mg/g)	G (h)	Y (%)
1	75	15	60	4	40	50	2.5	9.89
2	75	25	60	4	50	90	1.5	11.78
3	75	15	40	6	50	90	1.5	7.96
4	75	25	40	6	40	50	1.5	10.51
5	45	15	60	6	40	90	1.5	10.81
6	45	25	60	6	40	90	2.5	11.56
7	75	15	60	6	50	50	2.5	13.05
8	45	25	40	6	50	50	2.5	10.81
9	45	15	40	4	40	50	1.5	7.70
10	45	15	40	4	50	90	2.5	6.95
11	45	25	60	4	50	50	1.5	11.50
12	75	25	40	4	40	90	2.5	9.15

**Table 2:** Variance analysis of Plackett–Burman design

Source	Sum of squares	Degree of freedom	Mean square	*F*-value	*p* value
Regression model	35.31	7	5.04	7.69	0.0335
A	0.75	1	0.75	1.14	0.3449
B	6.64	1	6.64	10.13	0.0335
C	20.03	1	20.03	30.55	0.0052
D	4.99	1	4.99	7.61	0.0509
E	0.49	1	0.49	0.75	0.4341
F	2.29	1	2.29	3.49	0.1350
G	0.11	1	0.11	0.17	0.7006

#### Box–Benhnken design and data analysis

Using the basic of Plackett–Burman design, we selected the main optimization experimental factors and levels for analysis by Box–Benhnken response surface design and show the results in Table 3. Other factors levels that were used are sonication time of 60 min, a temperature of 45 °C, a cellulase concentration of 70 mg/g and an enzymatic hydrolysis time for 2 h.

**Table 3:** The factor coding and levels of the Box–Benhnken design

Factors	Real value	Coding	Levels		
			−1	0	1
C	X_1_	*x*_*1*_	40	50	60
B	X_2_	*x*_*2*_	15	20	25
D	X_3_	*x*_*3*_	4	5	6

*x*_*1*_ = (X_1_ − 50)*/*10; *x*_*2*_ = (X_2_ − 20)*/*5; *x*_*3*_ = (X_3_ − 5)*/*1

The analysis of variance for Box–Benhnken response surface design can be seen in Table 4. The regression equation for the extraction yield of flavonoids and the relevant analysis items are shown in the formula below:

**Table 4:** Observed and estimated values of Box–Benhnken response surface design

No.	C (%)	B (mL/g)	D	Y (%)	Estimated value (%)
1	1	1	0	12.66	12.60
2	0	0	1	13.02	12.65
4	−1	−1	0	10.58	10.69
5	1	1	0	11.95	11.84
6	0	0	0	13.92	14.13
7	0	0	0	13.38	14.13
8	−1	−1	0	12.05	12.10
9	1	1	−1	12.96	12.69
10	1	1	1	12.44	12.87
11	0	0	0	14.44	14.13
12	0	0	0	14.14	14.13
13	0	0	1	13.85	13.53
14	−1	−1	−1	12.09	11.67
15	0	0	−1	14.79	14.13
16	0	1	−1	12.22	12.60
17	0	−1	−1	12.50	12.82

$$\begin{aligned} {\text{Y}}\left( \% \right) = & - 5 5. 90 6 5 4 + 1. 7 3 5 5 9 {\text{X}}_{ 1} + 1. 7 3 800{\text{X}}_{ 2} + 2. 9 7 4 2 5 {\text{X}}_{ 3} - 0.0 10 8 7 2 {\text{X}}_{ 1} {\text{X}}_{ 2} \\ & - 9. 9 8 8 4 5 {\text{E}} - 00 3 {\text{X}}_{ 1} {\text{X}}_{ 3} + 0.0 5 5 2 4 4 {\text{X}}_{ 2} {\text{X}}_{ 3} \\ & - 0.0 1 4 2 6 8 {\text{X}}_{ 1}^{ 2} - 0.0 3 5 9 4 4 {\text{X}}_{ 2}^{ 2} - 0. 3 3 90 7 {\text{X}}_{ 3}^{ 2} \\ \end{aligned}$$

where Y is the extraction yield of flavonoids (%), X_1_ is the ethanol concentration (%), X_2_ is the liquid–solid ratio (mL/g) and X_3_ is the pH.

**Fig. 10:**
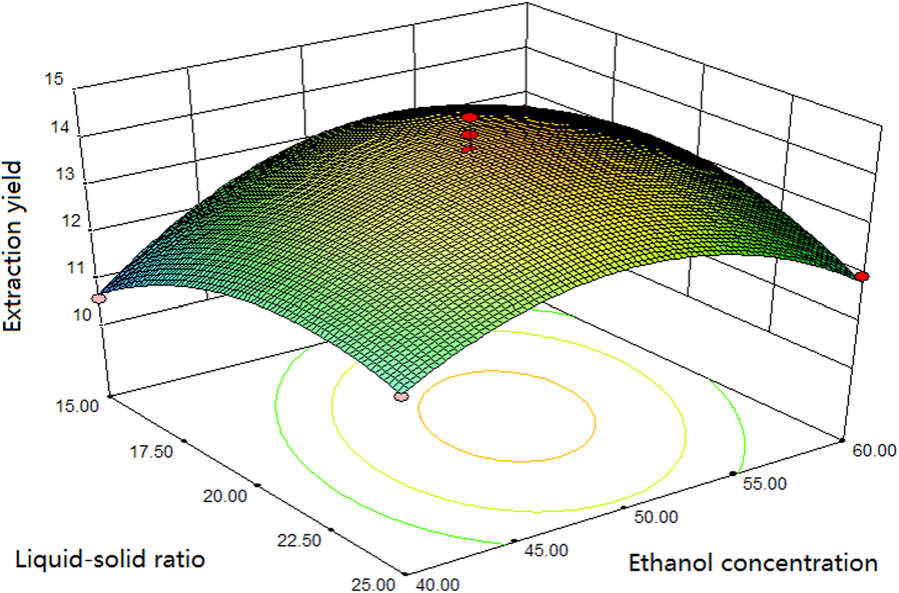
Response surface and contour plots showing the effect of ethanol concentration and liquid–solid ratio on the extraction yield

**Fig. 11:**
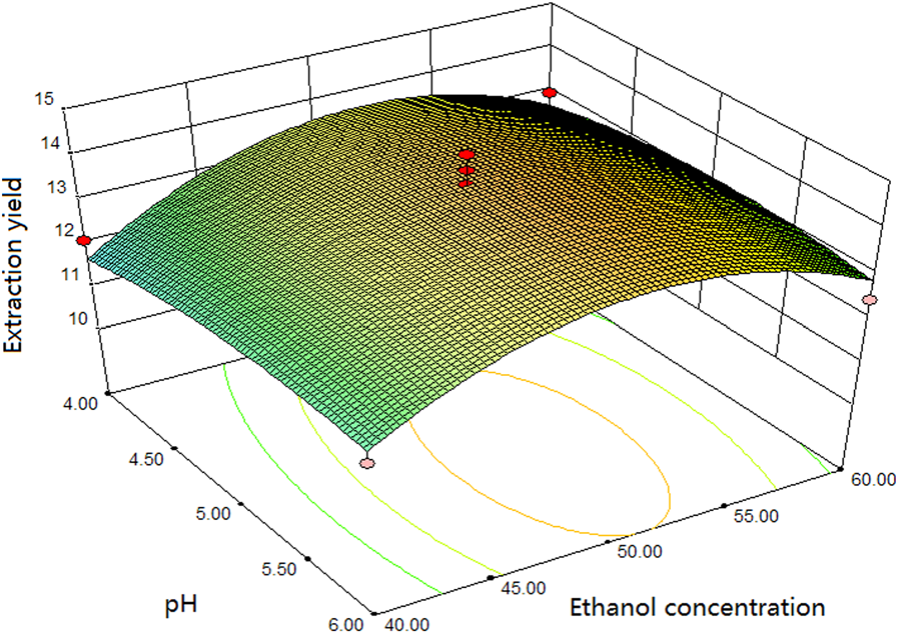
Response surface and contour plots showing the effect of ethanol concentration and pH on the extraction yield

**Fig. 12:**
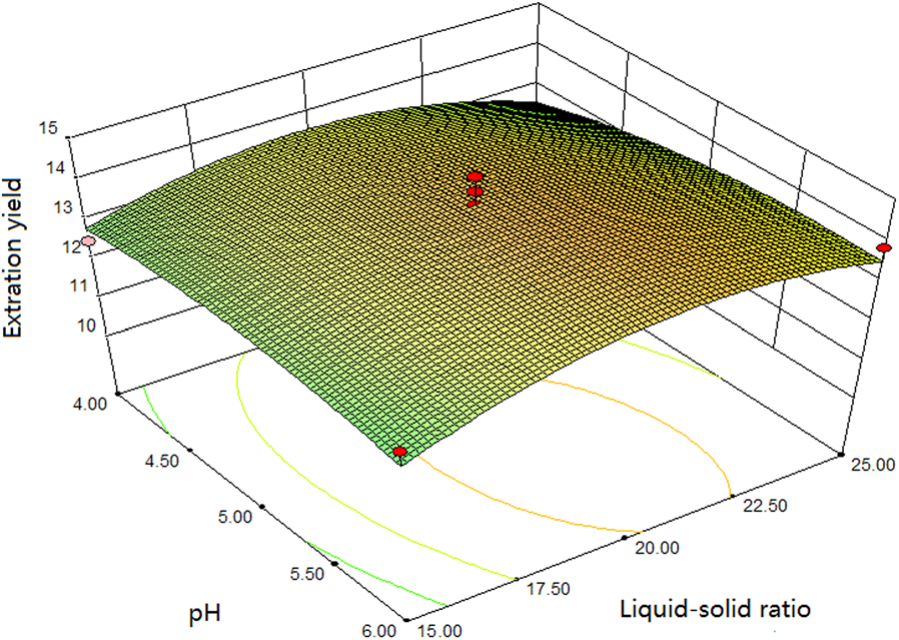
Response surface and contour plots showing the effect of liquid–solid ratio and pH on the extraction yield

The analysis of variance of the regression model were evaluated using the corresponding *F* and *p* values, and presented in Table 5. As shown in Table 5, the F value is calculated to be 6.05 and the p value is 0.0135, which suggests that the model is statistically significant. The model’s coefficient of determination (R^2^) is 0.9871, which indicates that more than 98.71 % of the response variability is explained by the model. The quadratic terms X_1_^2^ and X_2_^2^ are significant (*p* < 0.05), while, the interaction terms and linear terms are not significant (*p* > 0.05). This indicates that the extraction yield and relevant analysis items have obvious surface relationships, and interactions among each experimental factor are not significant. The lack of fit is not significant (*p* > 0.05), which means the regression equation may fit the actual situation. The response surface and contour plots of the ethanol concentration, liquid–solid ratio and enzymatic hydrolysis pH are shown in Figs. 10, 11 and 12.

**Table 5:** Variance analysis of regression equation for the extraction yield

Source	Sum of squares	Degree of freedom	Mean square	*F*-value	*p* value
Regression model	16.90	9	1.88	6.05	0.0135*
X_1_	1.37	1	1.37	4.42	0.0737
X_2_	0.22	1	0.22	0.70	0.4320
X_3_	0.29	1	0.29	0.92	0.3693
X_1_X_2_	1.18	1	1.18	3.81	0.0920
X_1_X_3_	0.04	1	0.04	0.13	0.7305
X_2_X_3_	0.31	1	0.31	0.98	0.3544
X_1_^2^	8.57	1	8.57	27.62	0.0012*
X_2_^2^	3.40	1	3.40	10.95	0.0129*
X_3_^2^	0.48	1	0.48	1.56	0.2519
Residual error	2.17	7	0.31		
Lack of Fit	1.04	3	0.35	1.23	0.4088
Pure error	1.13	4	0.28		
Total	19.08	16			
	R^2^ = 0.9871				

* Means significant (*p* < 0.05)

#### Results of response surface optimization and verification tests

The optimum extraction conditions via response surface optimization are as follows: the ethanol concentration, X_1_ = 51.14 %, the liquid–solid ratio, X_2_ = 20.52 mL/g, the enzymatic hydrolysis pH, X_3_ = 5.303, and the estimated optimal extraction yield is 14.20 %. The optimal experimental conditions were carried out on three repeated experiments to optimize and verify the reliability of the conditions used, and the levels of the other factors were as follows: sonication time was 60 min, enzymolysis temperature was 45 °C, enzyme concentration was 70 mg/g, enzymatic hydrolysis time was 2 h and the crushed mesh number was 0.355–0.85 mm. The test results are shown in Table 6.

**Table 6:** The results of the validation test

Test no.	1	2	3	Average	RSD %
Extraction yield (%)	14.87	14.09	15.33	14.76	4.24

The results of the validation test show that the RSD of the extraction yield is less than 5 %, and the average extraction yield is 14.76 %, which is near equivalent to the maximum extraction yield of 14.79 % seen in previous experiments listed in Table 4. It is concluded that this method is both effective and feasible.

Under the uniform extraction conditions of ultrasonic treatment 60 min, a liquid–solid ratio of 20.52 mL/g and an ethanol concentration of 51.14 %, the average of flavonoids extraction yield without enzyme assistance was 10.15 % (n = 3), while, with enzyme assistance this was increased to 14.76 % (n = 3) which is effectively a relative increase of 45.42 %.

## Conclusions

In this study, a regression model of cellulase-ultrasonic assisted extraction technology for flavonoids from *I. verum* residues was established. Using ethanol concentration, liquid–solid ratio and enzymatic hydrolysis pH as the independent variables, and the extraction yield of flavonoids as the dependent variable the optimum extraction process was determined. The concentration of ethanol is 51.14 %, the liquid–solid ratio is 20.52 mL/g, the enzymatic hydrolysis pH is 5.303, the sonication time is 60 min, the enzyme solution temperature is 45 °C, the amount of added enzyme is 70 mg/g, the enzymatic hydrolysis time is 2 h and the crushed mesh size is 0.355–0.85 mm. Under these optimum extraction conditions, the maximum flavonoids yield achieved is 14.76 %. Thus the data presented here indicate that the cellulase-ultrasonic assisted extraction technology has the potential be used for the industrial production of flavonoids from *I. verum*.
